# Evaluation of a program for routine implementation of shared decision-making in cancer care: results of a stepped wedge cluster randomized trial

**DOI:** 10.1186/s13012-021-01174-4

**Published:** 2021-12-29

**Authors:** Isabelle Scholl, Pola Hahlweg, Anja Lindig, Wiebke Frerichs, Jördis Zill, Hannah Cords, Carsten Bokemeyer, Anja Coym, Barbara Schmalfeldt, Ralf Smeets, Tobias Vollkommer, Isabell Witzel, Martin Härter, Levente Kriston

**Affiliations:** 1 Department of Medical Psychology, University Medical Center Hamburg-Eppendorf, Martinistrasse 52, 20246 Hamburg, Germany; 2 II. Department of Medicine, University Medical Center Hamburg-Eppendorf, Martinistrasse 52, 20246 Hamburg, Germany; 3 Department of Gynecology, University Medical Center Hamburg-Eppendorf, Martinistrasse 52, 20246 Hamburg, Germany; 4 Department of Oral and Maxillofacial Surgery, University Medical Center Hamburg-Eppendorf, Martinistrasse 52, 20246 Hamburg, Germany

**Keywords:** Shared decision-making, Implementation science, Cancer, Health services research, Stepped wedge design, Cluster randomized controlled trial, Outcome evaluation, Process evaluation

## Abstract

**Background:**

Shared decision-making (SDM) is preferred by many patients in cancer care. However, despite scientific evidence and promotion by health policy makers, SDM implementation in routine health care lags behind. This study aimed to evaluate an empirically and theoretically grounded implementation program for SDM in cancer care.

**Methods:**

In a stepped wedge design, three departments of a comprehensive cancer center sequentially received the implementation program in a randomized order. It included six components: training for health care professionals (HCPs), individual coaching for physicians, patient activation intervention, patient information material/decision aids, revision of quality management documents, and reflection on multidisciplinary team meetings (MDTMs). Outcome evaluation comprised four measurement waves. The primary endpoint was patient-reported SDM uptake using the 9-item Shared Decision Making Questionnaire. Several secondary implementation outcomes were assessed. A mixed-methods process evaluation was conducted to evaluate reach and fidelity. Data were analyzed using mixed linear models, qualitative content analysis, and descriptive statistics.

**Results:**

A total of 2,128 patient questionnaires, 559 questionnaires from 408 HCPs, 132 audio recordings of clinical encounters, and 842 case discussions from 66 MDTMs were evaluated. There was no statistically significant improvement in the primary endpoint SDM uptake. Patients in the intervention condition were more likely to experience shared or patient-lead decision-making than in the control condition (*d*=0.24). HCPs in the intervention condition reported more knowledge about SDM than in the control condition (*d* = 0.50). In MDTMs the quality of psycho-social information was lower in the intervention than in the control condition (*d* = − 0.48). Further secondary outcomes did not differ statistically significantly between conditions. All components were implemented in all departments, but reach was limited (e.g., training of 44% of eligible HCPs) and several adaptations occurred (e.g., reduced dose of coaching).

**Conclusions:**

The process evaluation provides possible explanations for the lack of statistically significant effects in the primary and most of the secondary outcomes. Low reach and adaptations, particularly in dose, may explain the results. Other or more intensive approaches are needed for successful department-wide implementation of SDM in routine cancer care. Further research is needed to understand factors influencing implementation of SDM in cancer care.

**Trial registration:**

clinicaltrials.gov, NCT03393351, registered 8 January 2018.

**Supplementary Information:**

The online version contains supplementary material available at 10.1186/s13012-021-01174-4.

Contributions to the literature

- This study evaluated a multi-component shared decision-making (SDM) implementation program that was informed by a pre-implementation pilot study (empirical foundation) and theoretically grounded in a conceptual framework.
- This study provides an example of a rigorous stepped wedge cluster randomized design that included a process evaluation and assessed implementation outcomes from various stakeholders’ perspectives.
- Process evaluation suggests that limited reach of several implementation strategies and a range of necessary adaptations that reduced fidelity could explain why the implementation program failed to facilitate uptake of SDM at the department level.

## Background

In cancer care, health care decisions often revolve around complex treatment options with various patterns of benefits and risks and with a substantial impact on the patient’s subsequent quality of life [1]. This makes it especially important to consider patients’ values and preferences during the decision-making process [2, 3]. Many patients with cancer prefer to be involved in medical decisions [4–7]. In shared decision-making (SDM), an important component of high-quality health care, patients and health care professionals (HCPs) build a team in the decision-making process by combining medical knowledge with personal preferences and values to find the option that best suits the patient’s individual situation [8–10]. Therewith, SDM is an important pillar of both evidence-based medicine and patient-centered care [11, 12]. SDM is widely supported by ethical considerations [13] and by health policy makers [14, 15]. A range of patient- and clinician-mediated interventions to facilitate SDM have been evaluated in clinical trials, including SDM communications skills training for HCPs [16] and patient decision aids (PtDAs [17],). However, translation into routine practice has repeatedly been found to be limited [4, 18–21]. This lack of implementation has been associated with patients’ decision regret as well as lower patient-reported quality of care and physician communication [7, 22].

In the past years, a range of SDM implementation efforts has been made. Some of these endeavors focused on the implementation of PtDAs as the main strategy to foster SDM implementation. Many of these studies did not explicitly ground their work in theoretical considerations [23], as recommended by implementation scientists [24, 25]. Several SDM implementation projects did include multiple strategies, e.g., the MAGIC (Making good decisions in collaboration) program in the UK [26] and an SDM implementation program in breast cancer care in the Netherlands [27]. In Germany, at the time of planning this study, no projects focusing on the implementation of SDM in routine clinical practice had been concluded [14, 28].

Building on the importance of using a theoretical underpinning in implementation projects [24, 25] and of conducting pre-implementation studies to understand the local context and its stakeholders’ perspectives on potential implementation strategies, we used the Consolidated Framework for Implementation Research (CFIR [29],) and developed a multi-component SDM implementation program for cancer care based on the results of a thorough pilot study. In this pilot study, we assessed the current state of SDM implementation and the needs of different stakeholders regarding SDM implementation at the same comprehensive cancer center that also participated in the implementation study reported here. The pilot study used a range of qualitative methods, including interviews, focus groups, and observational methods, and triangulated perspectives between different stakeholders and researchers. Detailed results are described elsewhere [18, 19, 30–32].

The aim of the present study was to evaluate this theoretically and empirically grounded multi-component program for implementation of SDM in routine cancer care.

## Methods

### Design

We used a stepped wedge design, a variant of the cluster randomized controlled trial, in which the participating clusters received the intervention in a randomized order. The SDM implementation program was sequentially introduced in each of the three participating departments in time intervals of 6 months, i.e., each department moved from control condition (prior to introduction of the implementation program) to intervention condition (exposure to implementation program). The findings are reported in accordance with relevant reporting guidelines ( [33, 34], see Additional files 1 and 2). Methodological details have been described in a published study protocol [28].

### Setting and participants

The study was conducted in three departments of a comprehensive cancer center within an academic hospital in Germany treating a wide range of cancer entities. Each department offers inpatient and outpatient care. We selected the departments due to their respective leadership’s high interest in the implementation of SDM identified in the pilot study, which is a known facilitator for SDM implementation [35]. The study team consisted of researchers with expertise in SDM interventions and implementation. Hospital administrators and health services managers were involved in the study in an advisory capacity (e.g., in a workshop meeting at the beginning of the study).

We aimed to include an unselected sample of patients who had a confirmed or suspected diagnosis of a neoplasm (ICD 10: C00-D49, excluding D10-D36), received health care at one of the participating departments, were 18 years old or older, and spoke German sufficiently. As it was not always possible to verify diagnosis and age at the time of recruitment, we decided to also include German-speaking patients with uncertainty regarding diagnosis or age who visited the cancer-specific in- and outpatient facilities at the departments during the data collection waves (see Additional file 3 for a list of changes from the study protocol). All physicians and nurses who were working at the departments at the time of the study were invited to participate.

### Intervention

The multi-component SDM implementation program was based on theoretical considerations [29] and empirical findings from a preparatory pilot study [18, 19, 30–32]. It consisted of SDM training for HCPs (one group session per HCP), individual SDM coaching of physicians (two sessions per physician), a patient activation intervention (i.e., Ask 3 Questions, ASK3Q [36, 37]), provision of information material and decision aids for patients, revision of quality management documents (i.e., incorporation of SDM in the departments’ standard operating procedures), and reflection on multidisciplinary team meetings (MDTMs) [28]. Which findings of the pilot study informed which component of the implementation program has been described in the study protocol [28]. While most strategies focused on the individual level (patient, HCP), the last two strategies focused on the organizational level. Additionally, we developed a title in laypeople’s terms and a label for this study that we used on all documents and on pens specifically designed for this study. As suggested by Proctor et al. [38], actors, actions, targets of action, temporality, and dose of each implementation strategy were defined a priori [28].

The control condition was standard medical decision-making without the specific implementation program to foster SDM. Although patient-centeredness has a continuously increasing impact on the organization of health care in Germany [14], specific effort to implement SDM in routine practice is generally absent. Therefore, the control condition did not include any intentional or direct SDM implementation efforts.

### Outcome evaluation

#### Measures and outcomes

Implementation outcomes [39] were collected from four sources: a standardized survey of patients, a standardized survey of HCPs, rating of audio-recorded clinical encounters, and systematic observation of MDMTs.

The primary outcome was uptake of SDM assessed by the 9-item Shared Decision Making Questionnaire (SDM-Q-9), a patient-reported measure of the SDM process in patient-physician encounters [40]. Secondary patient-reported outcomes included the uptake of SDM using the 3-item collaboRATE measure [41–43], a single-item measure of the experienced decision control during the rated clinical encounter (adapted Control Preference Scale (CPS) [44–46],), and a single-item measure of patient satisfaction.

HCP-rated measures were single items for self-assessed knowledge and use of SDM, a single-item measure of general preference for decision control in clinical encounters (adapted CPS [44]), the 8-item IcanSDM measure assessing perceived barriers of SDM implementation [47, 48] as an indicator of appropriateness of SDM, the 10-item Organizational Readiness for Implementing Change (ORIC) scale [49, 50], and six single-item assessments of acceptability of SDM adapted from McColl’s questionnaire on attitudes towards evidence-based practice [51] and the Evidence-Based Practice Attitude Scale (EBPAS [52],), and derived from results of the pilot study.

Outcomes for the audio-recorded clinical encounters were the uptake of SDM as measured by the Observer OPTION^5^ tool [53–55] and patient-rated single-item assessments of the experienced decision control in the rated encounter and the general preference for decision control in clinical encounters (adapted CPS [44],).

As indicators for penetration of SDM in MDTMs, for each case discussed in the MDTMs, observer-rated outcomes were the quality of information on patient view, the quality of psychosocial information, and the number of recommendations given, as measured by an adapted version of the Metric for the Observation of Decision Making in Multidisciplinary Team Meetings (MDT-MODe [32, 56],).

Most of these measures had been defined prior to starting the study [28] and all of them were specified prior to data analysis (for deviations from the study protocol regarding the outcome measures see Additional file 3). All surveys included assessment of demographic and clinical or professional information, respectively. Patients’ global health was assessed using a single item derived from the Short-Form-Health Survey (SF-12 [57],) and their distress was assessed using the German version of the NCCN Distress Thermometer [58].

#### Sample size considerations

In order to be able to identify a small to moderate effect (Cohen’s *d* of 0.3) of the implementation program on the patients’ experience of SDM, we aimed to collect data from 1440 patients [28]. The target sample size of HCPs was not fixed a priori (complete sampling). Additionally, we aimed to analyze the audio recordings of 144 clinical encounters (12 encounters × 3 departments × 4 measurement waves) and to observe 64 MDMTs (4 types of MDTMs × 4 meetings × 4 measurement waves).

#### Data collection

Data collection was planned at four measurement waves with a 2-month duration each. Some deviations from the study protocol occurred due to insufficient recruitment and, regarding the fourth measurement wave, the pandemic of the severe acute respiratory syndrome coronavirus 2 (SARS-CoV-2) (see Additional file 3). Hence, data were collected from baseline to month 2, months 9 to 10.5, months 17 to 18.5, and months 25 to 30 (including 10 weeks recruitment stop due to the pandemic).

Patients were approached by members of the study team in the waiting areas of outpatient clinics and on the wards for inpatients. They were transparently informed about the purpose of the study and gave written informed consent before participation in the paper and pencil survey and/or the audio recording of the clinical encounter with the physician. They were asked to fill out questionnaires anonymously after the clinical encounter. HCPs were approached in team meetings or via mail with the request to participate in the anonymous paper and pencil HCP survey. The ID for matching questionnaires from the same HCP at different measurement waves was created by the HCP and not decipherable by the study team. MDTMs were sampled from the four types of MDTMs for which the participating departments were responsible. MDTMs and consultations for audio recordings were selected unsystematically according to availability of staff resources during the measurement waves.

#### Data analysis

We used guidelines developed by the study team for data entry and quality control. Quality of quantitative data was checked by partial double entry and calculation of agreement rates. Quality of transcripts of audio recordings was examined through proofreading by a second member of the study team. For the evaluation of audio recordings members of the study team were trained in the use of the Observer OPTION^5^ measure by the principal investigator (IS), experienced in the method. Raters were blinded to the allocation of audio recordings regarding control and intervention condition.

For the outcome evaluation, an analysis plan was prepared and reported in the study protocol [28]. Analyses were performed consistently with the same statistical procedure for all outcomes, using linear mixed models for continuous and generalized linear mixed models for dichotomous outcomes [59]. All models included a fixed effect for the intervention, a linear fixed effect for the measurement time point (wave), and a random intercept for department differences [60]. All available cases were analyzed. Covariates were added to model deviations from the study protocol and trial registration, i.e., for returning questionnaires more than 14 days after the end of the respective measurement wave, for rating clinical encounters that took place more than 90 days before the survey, for underage patients, and for the patient’s diagnosis not being confirmed or suspected malignant neoplasm. Additional covariates, of which distribution were imbalanced between the intervention and control condition, were included as necessary in order to control confounding.

The last measurement wave of the study was impacted by the SARS-CoV-2 pandemic. We handled this incident in two ways [61, 62]. In the main analyses, an additional covariate was included to indicate measurement under pandemic conditions. In the per-protocol sensitivity analysis (see below), we considered data collected during the pandemic as missing. Estimated marginal means were calculated for the per-protocol population, i.e., participants fulfilling all per-protocol criteria and being surveyed under non-pandemic conditions.

We conducted several sensitivity analyses for the most important outcomes to test the robustness of the intervention effect estimate. In a “full-covariate” analysis, we fitted models including additional covariates to further minimize baseline imbalance. In a “categorical time” analysis, we allowed the wave effect to have a non-linear effect. In a “multiplicative effect” analysis, we tested whether including the interaction between intervention and wave influences the results. In a “heterogeneous effects” analysis, we included a department-level random slope for the intervention effect. In a “repeated measures” analysis, we took into account that a minority of the HCPs answered the survey more than once, leading to dependencies in the data. Finally, in a “per-protocol” analysis, we included only data that were collected in full accordance with the trial registration.

We have calculated standardized mean differences (Cohen’s *d*) by dividing the estimated mean difference between groups by the pooled observed standard deviation for continuous data [63] and by using approximations from the odds ratio in case of dichotomous data [64]. Findings with *P* < .05 were considered statistically significant. As the analyses of the secondary outcomes were not adjusted for multiple testing, these findings should be considered exploratory. All statistical analyses were conducted with IBM SPSS Statistics 25 (IBM Corp, Armonk, NY).

### Process evaluation

We assessed quantitative implementation process indicators (including reach) as well as fidelity and adaptations of the implementation strategies by systematic documentation on how the different strategies were implemented. Process evaluation was used to address necessary adaptations of the program throughout the study and to support the interpretation of the outcome evaluation results. Descriptive statistics were calculated to describe quantitative implementation process indicators and to assess reach of the different implementation strategies. Fidelity and adaptations were furthermore evaluated. The following dataset were analyzed: (a) structured field notes of observations made by the study team, (b) minutes of meetings with clinical partners, and (c) transcripts of process interviews with HCPs. Qualitative data analysis, using primarily a deductive approach, was conducted: First, a coding scheme was created by one researcher (HC). Second, approximately 25–30% of the material was coded (HC). Third, the coding scheme was discussed and revised within the study team (HC, PH, IS). Fourth, coded material was revised and the remaining material was coded (HC). Fifth and last, results were discussed in the study team (HC, PH, IS) and final revisions were made (HC).

## Results

### Outcome evaluation

#### Sample characteristics

**Fig. 1:**
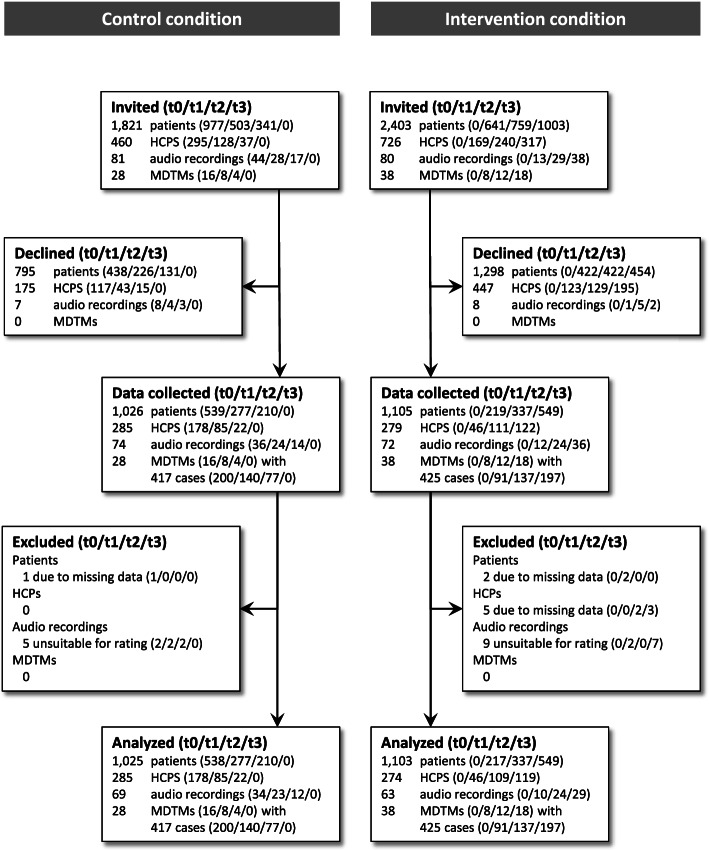
Case flow for patient survey, HCP survey, audio recordings, and MDTM observations

The case flow throughout the study is depicted in Figure 1. Return rates for analyzable patient surveys were 2128 of 4224 invited patients (50.4%). Most frequently voiced reasons for patients’ non-participation in the survey were prior participation in the study (*n* = 459), physical or psychological burden (*n* = 225), or no interest in the study (*n* = 183). 809 patients did not voice a reason for non-participation. 559 of 1186 potential HCP surveys were returned and included in data analyses (47.1%). 146 of 161 invited patients contributed to the study by allowing to audio-record their clinical encounter with a physician. Of these, 14 cases were not applicable for OPTION^5^ observer rating (i.e., both external raters appraised not applicable). This led to a return rate of analyzable audio recordings of 132 of 161 (82.0%).

On average, the 2128 surveyed patients (61.4% female, mean age 57.0 years) rated their level of subjective health and their level of distress as moderate. Most of them had a confirmed cancer diagnosis for less than five years. They visited the department mostly due to diagnostic, treatment-related, or monitoring reasons in approximately equal shares. About two-thirds reported that they were consulting their HCP about a treatment-related decision. Notable differences between intervention and control conditions were identified regarding gender, time since the first diagnosis, reason for visit, and the topic of decision. Table 1 gives a detailed overview on sample characteristics of surveyed patients.

**Table 1:** Patient sample characteristics

	Control (***n*** = 1025)	Intervention (***n*** = 1103)	Total (***n*** = 2128)
*Gender*, *n* (%)			
Female	739 (73.3)	543 (50.3)	1282 (61.4)
Male	265 (26.3)	536 (49.7)	801 (38.4)
Other or not specified	4 (0.4)	0 (0.0)	4 (0.2)
*Age*, mean (SD)			
Years	56.3 (16.2)	57.8 (15.7)	57.0 (15.9)
*Formal education*, *n* (%)			
Low ^a^	185 (18.6)	216 (20.2)	401 (19.4)
Intermediate ^b^	307 (30.8)	326 (39.5)	633 (30.7)
High ^c^	236 (23.7)	234 (21.8)	470 (22.7)
Very high ^d^	260 (26.1)	284 (26.6)	544 (26.3)
Other	8 (0.8)	9 (0.8)	17 (0.8)
*Occupational status* ^e^, *n* (%)			
(Self-)employed	442 (44.1)	476 (44.5)	918 (44.3)
Retired	433 (43.2)	470 (43.9)	903 (43.6)
Homemaker	61 (6.1)	44 (4.1)	105 (5.1)
Other ^f^ (< 5% each)	110 (11.0)	117 (10.9)	227 (11.0)
*Setting*, *n* (%)			
Inpatient	89 (8.7)	93 (8.4)	182 (8.6)
Outpatient	934 (91.3)	1010 (91.6)	1944 (91.4)
*Global health*, mean (SD)			
1 to 5, higher better	2.83 (0.85)	2.79 (0.85)	2.81 (0.85)
*Distress*, mean (SD)			
0 to 10, higher more	5.67 (2.48)	5.43 (2.45)	5.55 (2.47)
*Diagnosis*, *n* (%)			
Confirmed malignant neoplasm	759 (82.0)	826 (83.1)	1585 (82.6)
Suspected malignant neoplasm	11 (1.2)	9 (0.9)	20 (1.0)
In situ neoplasm or neoplasm of uncertain behavior	19 (2.1)	35 (3.5)	54 (2.8)
Benign neoplasm	30 (3.2)	11 (1.1)	41 (2.1)
Other diagnosis	107 (11.6)	113 (11.4)	220 (11.5)
*Time since initial diagnosis*, *n* (%)			
1 year or less	393 (47.9)	390 (42.0)	783 (44.8)
1 to 5 years	279 (34.0)	370 (39.9)	649 (37.1)
more than 5 years	148 (18.0)	168 (18.1)	316 (18.1)
*Reason for visit* ^e, g^, *n* (%)			
Diagnostic investigation	235 (23.5)	214 (20.0)	449 (21.7)
Initial communication of the diagnosis	165 (16.6)	158 (14.9)	323 (15.7)
Treatment planning	278 (27.9)	249 (23.4)	527 (25.6)
Treatment	167 (16.8)	239 (22.5)	406 (19.7)
Treatment monitoring	254 (25.5)	338 (31.8)	592 (28.8)
Aftercare	205 (20.6)	216 (20.3)	421 (20.4)
*Decision topic* ^e, g^, *n* (%)			
Diagnostic procedures	314 (33.1)	296 (29.2)	610 (31.1)
Surgery	304 (32.0)	257 (25.3)	561 (28.6)
Chemotherapy	207 (21.8)	379 (37.3)	586 (29.8)
Radiation therapy	69 (7.3)	101 (10.0)	170 (8.7)
Other treatment	100 (10.5)	99 (9.8)	199 (10.1)
*Surveyed in pandemic*, *n* (%)			
No	993 (100.0)	711 (66.7)	1704 (82.8)
Yes	0 (0.0)	355 (33.3)	355 (17.2)

*Notes.* Frequencies not adding up to the total number of participants within groups indicate missing data; percentages are calculated for valid data within the group

^a^low = no formal degree or graduation after less than 10 years at school

^b^intermediate = graduation after 10 or 11 years at school

^c^high = graduation after more than 11 years at school

^d^very high = college or university degree

^e^multiple choices possible

^f^including student/trainee, sick leave, parental leave, military service, unemployed

^g^only categories with more than 5% of the total sample are displayed

Among the 559 HCP responses (71.8% female, 67.9% 40 years old or younger), slightly more were from nurses compared to physicians. About 60% of HCPs had five years or more experience in oncological care, and the vast majority treated oncological patients at the time of the study. The responses were given by 408 different individuals, with 300 of them (73.5%) only participating once in the survey. No substantial differences were identified between the intervention and the control condition. For details on characteristics of surveyed HCPs, see Table 2.

**Table 2:** Health care professional sample characteristics

	Control (***n*** = 285)	Intervention (***n*** = 274)	Total (***n*** = 559)
*Gender, n* (%)			
Female	205 (73.4)	190 (69.9)	395 (71.8)
Male	68 (24.5)	78 (28.7)	146 (26.5)
Other or not specified	5 (1.8)	4 (1.4)	9 (1.6)
*Age*, *n* (%)			
30 years or younger	85 (30.7)	85 (31.6)	170 (31.1)
31 to 40 years	105 (37.9)	96 (35.7)	201 (36.8)
41 to 50 years	54 (19.5)	58 (21.6)	112 (20.5)
Older than 50 years	33 (11.9)	30 (11.2)	63 (11.5)
*Position*, *n* (%)			
Nurse	162 (58.1)	152 (56.9)	314 (57.5)
Junior physician	81 (29.0)	76 (28.5)	157 (28.8)
Senior or head physician	36 (12.9)	39 (14.6)	75 (13.7)
*Experience in oncology*, *n* (%)			
Less than 5 years	121 (44.5)	106 (39.6)	227 (42.0)
5 to 10 years	63 (23.2)	62 (23.1)	125 (23.1)
11 to 20 years	51 (18.8)	66 (24.6)	117 (21.7)
More than 20 years	37 (13.6)	34 (12.7)	71 (13.1)
*Currently works with cancer patients*, *n* (%)			
Yes	263 (94.3)	257 (95.2)	520 (94.7)
No	16 (5.7)	13 (4.8)	29 (5.3)
*Surveyed in pandemic*, *n* (%)			
No	285 (100.0)	155 (56.6)	440 (78.7)
Yes	0 (0.0)	119 (43.4)	119 (21.3)

*Notes.* Frequencies not adding up to the total number of participants within groups indicate missing data; percentages are calculated for valid data within the group

Audio recordings of 132 patients (56.5% female, mean age 58.6 years) were analyzed. The majority had a confirmed cancer diagnosis, which they received mostly less than five years ago. Notable group differences existed regarding gender, occupational status, diagnosis, time since first diagnosis, and appropriateness of the clinical encounter for SDM ratings as judged by external raters. Details on this sample are reported in Table 3.

**Table 3:** Audio-recorded consultations sample characteristics

	Control (***n*** = 69)	Intervention (***n*** = 63)	Total (***n*** = 132)
*Gender*, *n* (%)			
Female	49 (71.0)	24 (40.0)	73 (56.6)
Male	20 (29.0)	36 (60.0)	56 (43.4)
*Age*, mean (SD)			
Years	57.4 (14.1)	59.8 (15.8)	58.6 (14.9)
*Formal education*, *n* (%)			
Low ^a^	15 (25.4)	13 (24.1)	28 (24.8)
Intermediate ^b^	21 (35.6)	19 (35.2)	40 (35.4)
High ^c^	12 (20.4)	10 (18.6)	22 (19.5)
Very high ^d^	11 (18.6)	12 (22.2)	23 (20.4)
*Occupational status* ^e^, *n* (%)			
(Self-)employed	26 (44.8)	24 (45.3)	50 (45.0)
Retired	20 (34.5)	25 (47.2)	45 (40.5)
Homemaker	6 (10.3)	4 (7.5)	10 (9.0)
Other ^f^ (< 5% each)	7 (12.1)	3 (5.7)	10 (9.0)
*Cancer diagnosis*, *n* (%)			
Confirmed malignant neoplasm	54 (93.1)	46 (86.8)	100 (90.1)
Suspected malignant neoplasm	1 (1.7)	0 (0.0)	1 (0.9)
In situ neoplasm or neoplasm of uncertain behavior	0 (0.0)	1 (1.9)	1 (0.9)
Benign neoplasm	0 (0.0)	0 (0.0)	0 (0.0)
Other diagnosis	3 (5.2)	6 (11.3)	9 (8.1)
*Time since initial diagnosis*, *n* (%)			
1 year or less	21 (43.8)	18 (41.9)	39 (42.9)
1 to 5 years	17 (35.4)	19 (44.2)	36 (39.6)
More than 5 years	10 (20.8)	6 (14.0)	16 (17.6)
*Surveyed in pandemic*, *n* (%)			
No	69 (100.0)	34 (54.0)	103 (78.0)
Yes	0 (0.0)	29 (46.0)	29 (22.0)
*Doubts about appropriateness for rating* ^g^, *n* (%)			
No	56 (81.2)	41 (65.1)	97 (73.5)
Yes	13 (18.8)	22 (34.9)	35 (26.5)

*Notes.* Frequencies not adding up to the total number of participants within groups indicate missing data; percentages are calculated for valid data within the group

^a^low = no formal degree or graduation after less than 10 years at school

^b^intermediate = graduation after 10 or 11 years at school

^c^high = graduation after more than 11 years at school

^d^very high = college or university degree

^e^multiple choices possible

^f^including student/trainee, sick leave, military service, unemployed

^g^i.e., one of two external raters expressed doubts (if both raters expressed doubts, recording was excluded from analysis)

Sixty-six MDTMs with a total of 842 discussed cases were observed. This resulted in 425 cases in 38 meetings in the intervention condition, and 417 cases in 28 meetings in the control condition.

Across all data sources, approximately 20 to 25% of the data were collected during the SARS-CoV-2 pandemic. The pandemic situation began during the fourth (final) measurement wave of this study. At this point, all implementation intervals were completed, i.e., all three departments had moved to the intervention condition. Due to the stepwise implementation of the intervention, about 10% of the post-intervention patient data from department 1, about 40% of the post-intervention patient data from department 2, and about 90% of the post-intervention patient data from department 3 were collected during the pandemic situation.

#### Results of the outcome evaluation

We did not find a statistically significant difference between the control and intervention condition regarding the primary outcome, i.e., uptake of SDM from the patients’ perspective (as measured by the SDM-Q-9, Table 4).

**Table 4:** Results for the continuous outcomes

	Observed data						Estimated values				
	Control condition			Intervention condition			ICC	aMD	(95% ***CI***)	***P***	***d***
	***n***	mean	(SD)	***n***	mean	(SD)					
*Patient survey measures* ^a^											
Uptake of SDM (SDM-Q-9) ^b^	868	63.51	(26.40)	938	64.66	(26.87)	.026	0.56	(− 3.97 to 5.09)	.808	0.02
Uptake of SDM (collaboRATE) ^c^	954	83.07	(19.53)	1023	83.04	(18.91)	.018	1.23	(− 1.91 to 4.38)	.442	0.06
Satisfaction ^d^	992	3.40	(0.87)	1066	3.52	(0.74)	.006	0.09	(− 0.04 to 0.22)	.156	0.11
*HCP survey measures* ^e^											
SDM knowledge ^f^	279	3.53	(3.12)	273	6.21	(3.17)	.005	1.58	(0.61 to 2.54)	**.002**	0.50
SDM uptake ^f^	275	4.97	(2.65)	269	5.91	(2.49)	.035	0.37	(− 0.46 to 1.21)	.377	0.14
SDM barriers (IcanSDM) ^g^	281	4.50	(1.33)	272	4.46	(1.39)	.031	− 0.15	(− 0.59 to 0.30)	.521	0.11
Attitude: Patients initiating their active involvement foster SDM ^f^	280	7.47	(1.86)	271	7.56	(2.01)	.007	− 0.01	(− 0.62 to 0.60)	.976	− 0.01
Attitude: Positive that SDM gains importance ^f^	283	7.53	(1.76)	273	7.51	(1.97)	.027	0.07	(− 0.53 to 0.68)	.813	0.04
Attitude: Colleagues have positive attitudes towards SDM ^f^	282	6.22	(2.09)	271	6.15	(2.00)	.042	− 0.10	(− 0.76 to 0.56)	.766	− 0.02
Attitude: SDM is helpful in my routine care for patients ^f^	282	6.79	(1.73)	271	6.60	(2.04)	.009	− 0.35	(− 0.95 to 0.25)	.247	− 0.07
Attitude: SDM improves patient care ^f^	280	7.16	(1.88)	270	6.96	(2.05)	.015	− 0.13	(− 0.76 to 0.49)	.679	− 0.02
Attitude: Clinical experience more important than patient preferences ^f^	276	3.35	(2.40)	268	3.57	(2.38)	.007	0.09	(− 0.66 to 0.84)	.810	− 0.03
Organizational readiness for implementing SDM (ORIC) ^h^	279	3.44	(0.67)	272	3.43	(0.65)	.098	− 0.04	(− 0.25 to 0.18)	.731	− 0.06
Change commitment (ORIC subscale) ^h^	279	3.48	(0.71)	272	3.45	(0.70)	.089	− 0.06	(− 0.29 to 0.17)	.592	− 0.09
Change efficacy (ORIC subscale) ^h^	279	3.41	(0.70)	271	3.40	(0.70)	.081	− 0.02	(− 0.24 to 0.21)	.880	− 0.03
*Observer-rated audio recordings* ^i^											
Uptake of SDM (OPTION^5^) ^b^	69	21.30	(12.44)	63	16.71	(15.35)	.168	− 0.35	(− 8.26 to 7.57)	.931	− 0.03

*Notes. n* number of observations, *SD* standard deviation, *ICC* intraclass correlation coefficient, *aMD* adjusted mean difference, *CI* confidence interval, *P P*-value of the adjusted mean difference (intervention effect estimate), *d* Cohen’s *d* (pooled so that positive values indicate superiority of the intervention), HCP health care professional, *SDM* shared decision-making, *ORIC* Organizational Readiness for Implementing Change, *VAS* visual analog scale

^a^analyses adjusted for wave, gender, time since diagnosis, reason for visit, decision topic, protocol compliance of diagnostic status, protocol compliance of days since the rated consultation, protocol compliance of age, protocol compliance of the time point of answering, and pandemic situation

^b^sum scores, 0–100, higher values indicate more SDM

^c^sum scores, 1–100, higher values indicate more SDM

^d^1–4, higher values indicate higher satisfaction

^e^analyses adjusted for wave, protocol compliance of the time point of answering, and pandemic situation

^f^visual analog scale, 0–10, higher values indicate more knowledge/SDM/agreement with attitude

^g^0–10, higher values indicate stronger barriers

^h^1–5, higher values indicate more organizational readiness/commitment/efficacy

^i^analyses adjusted for wave, sex, time since diagnosis, occupational status, protocol compliance of diagnostic status, appropriateness of the recording for rating, and pandemic situation

Most secondary outcomes did not show a statistically significant difference between the groups either (Tables 4 and 5). Regarding the experienced decision control, patients in the intervention condition reported 55% higher odds of having had a shared or patient-led rather than a physician-led decision compared to the control condition (*P* = .017, *d* = 0.24; Table 5). HCPs in the intervention condition reported statistically significantly higher self-assessed knowledge about SDM (estimated difference 1.58 points on a 0 to 10 visual analog scale, *P* = .002, *d* = 0.50; Table 4). A detrimental effect was identified in terms of penetration of SDM in MDTMs. 58% lower odds of including the patient’s view appropriately in the MDTM case discussion were found in the intervention condition compared to the control condition (*P* = .020, *d* = − 0.48; Table 5). The sensitivity analyses largely confirmed these findings (Additional file 4).

**Table 5:** Results for the dichotomous outcomes

	Observed data				Estimated values				
	Control condition		Intervention condition		ICC	***aOR***	(95% ***CI***)	***P***	***d***
	***n/N***	(%)	***n/N***	(%)					
*Patient survey measures* ^a^									
Uptake of SDM (CollaboRATE topscore, yes vs. no)	294/954	(30.8)	277/1023	(27.1)	.020	0.97	(0.64 to 1.29)	.584	− 0.02
Decision control, adapted CPS (shared/patient vs. physician)	610/925	(65.9)	677/1006	(67.3)	.066	1.55	(1.08 to 2.22)	**.017**	0.24
*HCP survey measures* ^b^									
Control preference, adapted CPS (shared/patient vs. physician)	238/275	(86.5)	217/259	(83.8)	< .001	0.55	(0.23 to 1.15)	.178	− 0.33
*Patient report for audio recordings* ^c^									
Decision control, adapted CPS (shared/patient vs. physician)	39/51	(76.5)	42/51	(82.4)	.061	0.23	(0.03 to 1.70)	.148	− 0.81
Control preference, CPS (shared/patient vs. physician)	35/41	(85.4)	39/47	(83.0)	< .001	0.17	(0.02 to 1.63)	.122	− 0.98
*Observation of MDTMs* ^d^									
Information on patient view (substantial vs. less)	78/416	(18.8)	72/424	(17.0)	< .001	1.17	(0.67 to 2.04)	.584	0.09
Psychosocial information (substantial vs. less)	51/416	(12.3)	33/424	(7.8)	.004	0.42	(0.20 to 0.87)	**.020**	− 0.48
Multiple options recommended (yes vs. no)	14/364	(3.8)	8/368	(2.2)	< .001	0.82	(0.30 to 2.27)	.704	− 0.11

*Notes. n* number of observations with events, *N* number of total observations, *ICC* intraclass correlation coefficient, *aOR* adjusted odds ratio, *CI* confidence interval, *P P*-value of the adjusted odds ratio (intervention effect estimate), *d* Cohen’s *d* (pooled so that positive values indicate superiority of the intervention), *HCP* health care professional, *SDM* shared decision-making, *CPS* control preference scale, *MDTM* multidisciplinary team meeting

^a^analyses adjusted for wave, gender, time since diagnosis, reason for visit, decision topic, protocol compliance of diagnostic status, protocol compliance of days since the rated consultation, protocol compliance of age, protocol compliance of the time point of answering, and pandemic situation

^b^analyses adjusted for wave, protocol compliance of the time point of answering, and pandemic situation

^c^analyses adjusted for wave, sex, time since diagnosis, occupational status, protocol compliance of diagnostic status, appropriateness of the recording for rating, and pandemic situation

^d^analyses adjusted for wave and pandemic situation

Intraclass correlations were below 5% in most cases, with the largest between-department variations regarding organizational readiness for implementing change, observer-assessed uptake of SDM, and patient-reported decision control (Tables 4 and 5).

### Process evaluation

296 pages of field note documentation, minutes of 39 meetings, and 107 process interviews with 126 participants were analyzed.

Reach was calculated for two of the implementation strategies: SDM trainings for HCPs and individual coaching for physicians. Overall, 173 of 392 eligible HCPs (44%) participated in an SDM training. Fewer eligible nurses participated in comparison to eligible physicians (41% nurses, 52% physicians). Over all three departments, 57 of 118 eligible physicians (48%) participated in at least one coaching session. 37 of 118 (31%) participated in both coaching sessions. There was considerable variation with regards to the participation rates between departments, especially for SDM training (range: 35 to 73%).

Over the course of the three implementation intervals, 2709 postcards of the patient activation intervention ASK3Q, 762 information brochures “Patienten und Ärzte als Partner” (English: patients and physicians as partners [65],) and 370 generic decision aids [66, 67] were distributed to the departments. Furthermore, 136 ASK3Q posters were hung. For more detailed information on reach and implementation indicators, see Additional file 5.

Concerning fidelity, several adaptations were made regarding the different implementation strategies. Most adaptations concerned dose and temporality of the implementation strategies. For example, the SDM team trainings for HCPs lasted on average 50 minutes instead of two hours as planned a priori. Also, some coaching sessions took place without prior training of physicians. Table 6 gives an overview on how the strategies were originally planned in the study protocol [28] and which adaptations were made.

**Table 6:** Fidelity and adaptations

		SDM training for HCPs	Individual coaching for physicians	Patient activation strategy	Provision of patient information material and decision aids	Revision of the department’s quality management documents	Critical reflection of current organization of MDTMs
**Actor(s)**	*Planned*	Trained HCPs of respective department (trained by research team in a train-the-trainer workshop), research team	Research team	Clinical staff and research team	Clinical staff and research team	Research team, quality management department, and head HCPs of each department	Clinical staff and research team
	*Adaptations*	Research team led the trainings, trained HCPs less active than planned	No adaptations	Research team was main driver of dissemination	Research team was main driver of dissemination	Not all targeted actors from departments participated	Partly expansion of targeted actors to additional departments involved in the respective MDTMs
**Action(s)**	*Planned*	Interdisciplinary SDM training for physicians and nurses	Participant observation of physician-patient interaction and provision of feedback	Dissemination of material encouraging patients to ask questions regarding treatment options	Dissemination and use of information material and decision aids	Inclusion of SDM in quality management documents	Meetings with respective head of department and members of the clinical teams responsible for the MDTMs
	*Adaptations*	Only 32% of trainings were interdisciplinary; non-participants received training material by mail	Sometimes limited realm of feedback due to encounters without decision-making	Additional dissemination on department websites	Additional dissemination on department websites; lack of decision-specific patient decision aids in German	Additional development of a stand-alone quality management document on SDM	Head of department 2 did not participate
**Target(s) of action**	*Planned*	HCPs working at respective department	HCPs working at respective department	Patients being treated in respective department	Patients being treated in respective department	All staff working at respective department	All patient cases discussed in MDTMs
	*Adaptations*	No adaptations	No adaptations	No adaptations	No adaptations	No adaptations	No adaptations
**Temporality**	*Planned*	Beginning of implementation phase in respective department	First coaching should be within 4 weeks after training	Throughout implementation phase in respective department with start at beginning of phase	Throughout implementation phase in respective department with start after HCP training	Beginning of implementation phase in respective department	Throughout implementation phase in respective department
	*Adaptations*	No adaptations	Some coaching sessions delayed or without prior training	No adaptations	No adaptations	Delayed start, expansion of time frame	Expansion of time frame
**Dose**	*Planned*	Two hours training	Two coaching sessions with oral and written feedback per HCP	Initial set up of material in different department areas, need-based restocking	Initial set up of material in different department areas, need-based re-stocking	Short oral presentation of new documents in team meetings, combined with email to staff members	Two to three meetings of approx. 60 min per department
	*Adaptations*	Mean duration of team trainings 49.62 minutes	Partly only one coaching session	No adaptations	No adaptations	Oral presentations did not take place	Less meetings than planned (*n* = 5 in total)

*Notes. SDM* shared decision-making, *HCPs* health care professionals, *MDTMs* multidisciplinary team meetings

## Discussion

In this large-scaled study, the results on the primary and most secondary outcomes imply that the introduction of a multi-component program did not lead to more SDM implementation in the implementation condition compared to the control condition. Limited positive effects were found on few secondary outcomes, including an increased knowledge on SDM in HPCs. Results of the process evaluation yielded limited reach, and considerable adaptations of some of the implementation strategies were required.

Results from a pilot study formed the empirical basis of the implementation program. The pilot study led to a pre-selection of participating departments based on their head physicians being open to SDM and to the implementation study. The thorough prior analysis of the current state allowed the study team to get familiar with the respective setting and local barriers for SDM implementation before the beginning of the implementation trial. Needs identified during the pilot study were largely incorporated in the implementation program [28]. However, some aspects could not be considered as intended. Interdisciplinary training and facilitating team communication were planned but could be accomplished to a limited extent only [19, 31]. Also, the implementation program had a focus on HCPs. Patient empowerment training, tailored patient decision aids, and the establishment of a patient advocate were not included in the implementation program [31].

According to the National Cancer Institute [68] and the Consolidated Framework for Implementation Research [29], certain changes to core components of implementation strategies, e.g., reduction of dosage, which was necessary for several of the strategies in this study, have to be considered “red light changes” that should be avoided. While we deemed those adaptations necessary to fit the local context (due to limited available resources in the departments), they might have undermined the effectiveness of the program. In retrospect, the study might have benefitted from more rigorous and critical discussion of the possible advantages and disadvantages of adaptations. In this context, it is important to discuss the aspect of stakeholder engagement within the participating departments. Implementation science recommends to reflect a priori on capacities and resources within an organization [68]. This has not been in the focus of this study and might have led to implementation strategies that did not match the existing capacities. Thus, it was difficult for the core implementation team to balance fidelity and adaptations, leading to these potentially critical changes. The process evaluation also showed limited reach, suggesting that even a program with lower dose than initially planned was difficult to implement in the participating departments. Future implementation studies might benefit from more detailed a priori planning of resource allocation together with clinical leaders. Furthermore, despite controversial views, financial reimbursement for SDM or payment models incentivizing SDM might ease resource allocation to foster SDM implementation [35, 69].

Also, we have to reflect critically, whether the implementation strategies addressed attitudes and beliefs of HCPs enough [68], which has been found a key factor in SDM implementation in a multicenter study from the UK [26]. The implementation program had an effect on HCPs’ SDM knowledge, but not on their attitudes towards SDM. Even though attitudes towards SDM were found to be relatively positive, there was considerable variation between HCPs. Also, positive individual attitudes reported in a survey might not suffice to implement SDM behaviors in routine care. Future research could incorporate stakeholder engagement and participatory research ( [70], e.g., co-design of implementation strategies) as well as performance feedback ( [71], e.g., direct patient-reported feedback for HCPs regarding SDM) as potential means to foster the translation into routine SDM behavior.

Regarding patient decision aids and information material, it was not part of this trial to develop new decision aids as other implementation studies or studies with hybrid effectiveness-implementation designs [72] do (e.g. [73],). When systematically screening for evidence-based decision aids in German language for cancer-related decisions within this trial, the lack of such material became apparent. Instead we distributed a generic patient decision aid [66, 67]. Thus, this strategy has probably not developed its full strength. Furthermore, while we were able to document the amount of material distributed, we were not able to assess reach of this strategy as well as of the ASK3Q strategy.

Despite being seen as important in the pre-implementation study [30–32], our implementation strategy targeting MDTMs did not lead to structural changes on the organizational level. Limited capacities, resources, and stakeholder engagement within the departments that prioritize such changes might explain the lack of effects on MDTMs. Yet, in light of recent literature on barriers to SDM implementation [36, 69], it can be assumed that SDM implementation at the department level is not possible without a range of organizational changes that eventually—together with changes on the individual level—lead to a culture or paradigm shift. The revision of quality management documents alone was most probably not enough. Organizational changes could include mandatory documentation of psychosocial patient information and patient preferences in the electronic medical record as well as standardized integration of these aspects in MDTMs. While a range of strategies to foster SDM on the organizational level have been suggested in the literature [35], the lack of evidence of their effectiveness limits leverage to convince the highest level leadership to support such changes. A long-term US implementation project has shown that developing an organizational culture receptive to SDM uptake can take years [74]. Thus, the length of our program, limited by the total funding period of three years, could have been too short. Also, factors that have been found to influence SDM implementation at the level of health systems (e.g., payment models, medical education [35], cannot be changed by an implementation program like ours.

Although we did not find convincing evidence of an average positive effect of the implementation program in the whole study population, it is possible that considerable positive (or negative) effects are present in certain subgroups (e.g., physicians vs. nurses), contexts, or settings. The distal evaluation of effects at the department level (instead of individual HCP level) was not designed to detect potential changes of SDM behavior of individual HCPs. In further analyses, we will explore whether a heterogeneity of effects exists and, if so, how it can be explained [75].

The results of this study can be compared to several other very recent SDM implementation studies. Another German study evaluating a large-scale SDM implementation program found statistically significant effects for their multi-component implementation program in an interim analysis with data from a single department [76]. This trial used an uncontrolled before-and-after design and a different primary outcome measure to assess uptake of SDM [77]. The difference in results could be attributed to a much higher reach with over 90% of physicians from that department participating in an SDM training. It can also be explained by the strong involvement of the clinical team in the creation of new decision aids for their department and the temporary allocation of workforce resources to the study [76]. An SDM implementation trial for breast cancer care in the Netherlands found effects on observer-assessed SDM (assessed with OPTION^5^), but not with regards to the patient-assessed SDM (assessed with the SDM-Q-9) [27]. This study used an unpaired before-and-after study design and only included patients with breast cancer facing a treatment decision. Furthermore, the Dutch implementation program was co-designed with each participating clinic, allowing for major adaptations regarding focus and content of the implementation efforts [27]. This might have increased stakeholder engagement. The comparison of these recent implementation studies might bring valuable insights into what works and what does not work regarding the implementation of SDM in routine care.

### Strengths and limitations

A central strength of this trial is its high ecological validity. We managed to investigate a largely unselected population of patients, HCPs, clinical encounters, and MDTMs, which were representative of routine care in three departments of a German comprehensive cancer center. The study was informed by a pre-implementation pilot study and theoretically grounded in a conceptual framework. Also, the thorough execution of the study protocol including an extensive process evaluation is a major strength of this trial. Its further advantages include its large sample size and statistical power, the investigation of a wide range of outcomes from several perspectives, and a careful examination of the robustness of the findings.

As this study was a single-center trial, caution is necessary regarding generalizability of its results. Furthermore, only three clusters were included, which might limit the applicability of the stepped wedge cluster randomized design. Additionally, while the study was carried out according to schedule until the last measurement wave, the SARS-CoV-2 pandemic led to a delay in completing the final measurement wave. The pandemic situation had a considerable impact on routine healthcare (e.g., more remote consultations, restrictions to MDTMs, time constraints of HCPs [78, 79],) and might have impacted several of our implementation strategies (e.g., no training to apply SDM in remote consultations). Hence, the pandemic situation was incorporated as a covariate in the data analyses.

## Conclusion

In the present study, we did not find a statistically significant increase in the average level of SDM as perceived by the patients by applying an empirically and theoretically grounded multi-component implementation program to foster SDM in cancer care. Limited reach and considerable adaptations might explain the lack of change. As prior work suggested [23], there “are many miles to go” to fully implement SDM in routine practice. Future work should investigate other or more intensive approaches for successful department-wide implementation of SDM in routine cancer care and further assess factors influencing implementation of SDM in cancer care.

## Supplementary Information

**Additional file 1.** Completed Standards for Reporting Implementation Studies (StaRI) checklist.

**Additional file 2.** Completed checklist of information to include when reporting a stepped wedge cluster randomised trial (SW-CRT).

**Additional file 3.** Methodological changes from the study protocol.

**Additional file 4.** Sensitivity analyses.

**Additional file 5.** Reach and further implementation indicators.

## Data Availability

Deidentified data that support the findings of this study are available on reasonable request. Investigators who propose to use the data have to provide a methodologically sound proposal directed to the corresponding author. Signing a data use/sharing agreement will be necessary, and data security regulations both in Germany and in the country of the investigator who proposes to use the data must be complied with. Preparing the data set for use by other investigators requires substantial work and is thus linked to available or provided resources.

## Abbreviations

AC: Anja Coym
AL: Anja Lindig
ASK3Q: Ask 3 Questions
BS: Barbara Schmalfeldt
CB: Carsten Bokemeyer
CFIR: Consolidated Framework for Implementation Research
CPS: Control Preference Scale
EBPAS: Evidence-Based Practice Attitude Scale
HC: Hannah Cords
HCPs: Health care professionals
IS: Isabelle Scholl
IW: Isabell Witzel
JZ: Jördis Zill
LK: Levente Kriston
MAGIC: Making good decisions in collaboration
MDT-MODe: Metric of the Observation of Decision Making in Multidisciplinary Team Meetings
MDTMs: Multidisciplinary team meetings
MH: Martin Härter
ORIC: Organizational Readiness for Implementing Change
PH: Pola Hahlweg
PtDAs: Patient decision aids
RS: Ralf Smeets
SARS-CoV-2: Severe acute respiratory syndrome coronavirus 2
SDM: Shared decision-making
SDM-Q-9: 9-item Shared Decision Making Questionnaire
TV: Tobias Vollkommer
WF: Wiebke Frerichs
