# Supplementary material for: Evaluation of a program for routine implementation of shared decision-making in cancer care: results of a stepped wedge cluster randomized trial
Source: Implement Sci. 2021 Dec 29;16:106. doi: 10.1186/s13012-021-01174-4 (PMC8715412; doi:10.1186/s13012-021-01174-4)
Supplement: Supplementary file 3 — Additional file 3. Methodological changes from the study protocol. [file 13012_2021_1174_MOESM3_ESM.pdf]

Additional file 3. Methodological changes from the study protocol.

Domain	Study protocol / trial registration	Realization	Rationale
Patient inclusion criteria	Patients with suspected or diagnosed neoplasms (ICD 10: C00-D49; excluding D10-D36)	Primary analysis: inclusion of all patients receiving care in the participating departments, including also patients without neoplasm, with benign neoplasm, and missing data on diagnosis;<br>Per protocol analysis: inclusion of patients with a suspected or confirmed neoplasm (excluding benign neoplasms).	The implementation program aimed to influence decision-making generally in every health care aspect of the participating departments without being selective. A per protocol analysis was performed as a sensitivity analysis to check the effects of this change. The restriction to suspected or confirmed malignant neoplasms was made due to the expectation that diagnostic procedures, treatments, course of disease, and medical decisions for malignant neoplasms are fundamentally different to those for benign neoplasms.
Patient inclusion criteria	Confirmed age of 18 years or older	Primary analysis: inclusion of patients of any age receiving care in the participating departments, including patients under 18 years and with missing data on age;<br>Per protocol analysis: inclusion of patients 18 years and older.	The implementation program aimed to influence decision-making generally in every health care aspect of the participating departments without being selective. A per protocol analysis was performed as a sensitivity analysis to check the effects of this change.
Outcome measures	See published study protocol	Omitted: penetration assessed using routine data from quality assurance patient experience surveys.	Data for this secondary outcome were not available, because the patient experience surveys by the Office for Quality Management and Clinical Process Management were paused and changed during the study due to external reasons.
Outcome measures	See published study protocol	Added: patient-rated decision control and satisfaction; HCP-rated knowledge, uptake of SDM and control preference; additional HCP-rated SDM acceptability items.	These secondary outcomes were added in order to gain additional insights on the mechanisms and effects of implementation.
Measurement waves	Four measurement waves, two months each at months 1/2, 9/10, 17/18, and 25/26	Minor adjustments in temporality in the second and third wave (months 9-10.5, months 17-18.5);<br>Major adjustment in fourth wave (months 25 to 30, including ten weeks recruitment stop).	Measurement waves were expanded for up to two weeks to reach sufficiently large data sets (see main text – methods – measures and outcomes for planned sample sizes). Due to the pandemic of the severe acute respiratory syndrome coronavirus 2 (SARS-CoV-2), data collection in the fourth measurement wave had to be stopped for approximately ten weeks. Once it was possible to restart data collection, the research team needed to take the following precautions: weekly SARS-CoV-2 testing, use of personal protective gear, regular disinfection of pens and clipboards, presence of only one member of the study team in multidisciplinary team meetings. Nevertheless, data could be collected from all departments in sufficient amount.
