# Supplementary material for: Evaluation of a program for routine implementation of shared decision-making in cancer care: results of a stepped wedge cluster randomized trial
Source: Implement Sci. 2021 Dec 29;16:106. doi: 10.1186/s13012-021-01174-4 (PMC8715412; doi:10.1186/s13012-021-01174-4)
Supplement: Supplementary file 4 — Additional file 4. Sensitivity analyses. [file 13012_2021_1174_MOESM4_ESM.pdf]

Additional file 4. Sensitivity analyses.

	Original analysis	Sensitivity analyses					
		Full covariate analysis	Categorical time analysis	Multiplicative effect analysis	Heterogeneous effects analysis	Repeated measures analysis	Per protocol analysis
	***PE* (95% *CI*)**	***PE* (95% *CI*)**	***PE* (95% *CI*)**	***PE* (95% *CI*)**	***PE* (95% *CI*)**	***PE* (95% *CI*)**	***PE* (95% *CI*)**
Patient survey measures							
Uptake of SDM (SDM-Q-9)	0.56 (-3.97 to 5.09)	2.03 (-2.71 to 6.77)	1.14 (-3.48 to 5.77)	2.54 (-4.91 to 9.99)	0.56 (-3.97 to 5.09)	N/A	1.91 (-4.14 to 7.97)
Uptake of SDM (CollaboRATE)	1.23 (-1.91 to 4.38)	2.56 (-0.82 to 5.95)	1.35 (-1.85 to 4.55)	2.06 (-3.16 to 7.27)	1.23 (-1.91 to 4.38)	N/A	2.46 (-1.92 to 6.83)
Decision control (adapted CPS)	1.55* (1.08 to 2.22)	1.68* (1.13 to 2.50)	1.57* (1.09 to 2.27)	1.72* (1.01 to 2.94)	1.55* (1.08 to 2.22)	N/A	1.60 (0.98 to 2.61)
HCP survey measures							
SDM knowledge	1.58** (0.61 to 2.54)	1.75*** (0.87 to 2.63)	1.62** (0.65 to 2.59)	0.36 (-1.80 to 2.52)	1.55* (0.34 to 2.77)	2.63*** (1.99 to 3.26)	1.63** (0.58 to 2.69)
SDM uptake	0.37 (-0.46 to 1.21)	0.38 (-0.41 to 1.18)	0.35 (-0.48 to 1.19)	0.72 (-1.11 to 2.54)	0.27 (-1.19 to 1.73)	0.92** (0.39 to 1.45)	0.62 (-0.31 to 1.54)
Organizational readiness (ORIC)	-0.04 (-0.25 to 0.21)	-0.04 (-0.25 to 1.18)	-0.04 (-0.25 to 0.18)	-0.28 (-0.74 to 0.19)	-0.04 (-0.41 to 0.32)	0.06 (-0.08 to 0.19)	0.07 (-0.17 to 0.30)
Observer-rated audio-recordings							
Uptake of SDM (OPTION[5])	-0.35 (-8.26 to 7.75)	0.07 (-9.55 to 9.69)	-0.41 (-8.34 to 7.53)	-1.27 (-18.3 to 15.76)	-0.15 (-9.79 to 9.49)	N/A	3.54 (-6.54 to 13.61)
Observation of MDTMs							
Information on patient view	1.17 (0.67 to 2.04)	N/A	1.23 (0.69 to 2.17)	0.64 (0.21 to 1.97)	1.17 (0.67 to 2.04)	N/A	1.17 (0.68 to 2.01)

Notes. PE = parameter estimate; *CI* = confidence interval; SDM = shared decision-making; CPS = Control Preference Scale; HCP = health care professional; ORIC = Organizational Readiness for Implementing Change; MDTM = multidisciplinary team meeting; * P< 05; ** P<.01; *** P<.001.
