# Supplementary material for: Evaluation of a program for routine implementation of shared decision-making in cancer care: results of a stepped wedge cluster randomized trial
Source: Implement Sci. 2021 Dec 29;16:106. doi: 10.1186/s13012-021-01174-4 (PMC8715412; doi:10.1186/s13012-021-01174-4)
Supplement: Supplementary file 5 — Additional file 5. Reach and further implementation indicators. [file 13012_2021_1174_MOESM5_ESM.pdf]

Additional file 5. Reach and further implementation indicators.

	Department 1		Department 2		Department 3		Total	
SDM training for HCPs								
Eligible, n								
Physicians	67		25		26		118	
Nurses	153		60		61		274	
Total	220		85		87		392	
Attending, n (% of eligible)								
Physicians	28	(41.8)	20	(80.0)	13	(50.0)	61	(51.7)
Nurses	50	(32.7)	42	(70.0)	20	(32.8)	112	(40.9)
Total	78	(35.4)	62	(72.9)	33	(37.9)	173	(44.1)
Individual SDM coaching of physicians								
Eligible, n	67		25		26		118	
Attending, n (% of eligible)								
First coaching	29	(43.3)	15	(60.0)	13	(50.0)	57	(48.3)
Second coaching	19	(28.4)	8	(32.0)	10	(38.5)	37	(31.4)
Observed encounters per coaching, mean (range)	4.0	(1-10)	3.7	(1-10)	3.0	(1-7)	3.7	(1-10)
Weeks between training and first coaching, mean (range)	8.4	(0-42)	14.6	(3-49)	9.2	(2-20)	10.7	(0- 49)
Coaching without prior training, n (% of eligible)	12	(41.4)	2	(13.3)	6	(46.2)	20	(35.1)
Patient activation intervention: Ask 3 Questions								
Distributed postcards, n	1,188		943		578		2,709	
Distributed posters, n	47		59		30		136	
Provision of information material and decision aids for patients								
Distributed brochures "Patients and physicians as partners", n	485		126		151		762	
Distributed Ottawa Personal Decision Guides, n	179		92		99		370	
Revision of quality management documents								
Number of revised documents, n	3		2		8		13 [a]	
Reflection on multidisciplinary team meetings								
Number of meetings, n	3 [b]		2 [b]		1		5 [b]	

Notes: n = number; [a] Additionally one new stand-alone quality management document on SDM was developed; [b] One meeting was a joint meeting between clinics 1 and 2.
